# Nomogram to predict postoperative complications after cytoreductive surgery for advanced epithelial ovarian cancer: A multicenter retrospective cohort study

**DOI:** 10.3389/fonc.2022.1052628

**Published:** 2022-11-23

**Authors:** Caixia Jiang, Yingwei Liu, Junying Tang, Zhengyu Li, Wenjiao Min

**Affiliations:** ^1^ Department of Obstetrics and Gynecology, West China Second University Hospital, Sichuan University, Chengdu, China; ^2^ Department of Obstetrics and Gynecology, The First Affiliated Hospital of Chongqing Medical University, Chongqing, China; ^3^ Psychosomatic Department, Sichuan Provincial People’s Hospital, University of Electronic Science and Technology of China, Chinese Academy of Sciences Sichuan Translational Medicine Research Hospital, Chengdu, China

**Keywords:** advanced epithelial ovarian cancer, postoperative complications, cytoreductive surgery, nomogram, predict

## Abstract

**Objective:**

To establish nomograms to predict the risk of postoperative complications following cytoreductive surgery in patients with advanced epithelial ovarian cancer (AEOC).

**Methods:**

A multicenter retrospective cohort study that included patients with FIGO stage IIIC-IV epithelial ovarian cancer who underwent cytoreductive surgery was designed. By using univariate and multivariate analyses, patient preoperative characteristics were used to predict the risk of postoperative complications. Multivariate modeling was used to develop Nomograms.

**Results:**

Overall, 585 AEOC patients were included for analysis (training cohort = 426, extrapolation cohort = 159). According to the findings, the training cohort observed an incidence of postoperative overall and severe complications of 28.87% and 6.10%, respectively. Modified frailty index (mFI) (OR 1.96 and 2.18), FIGO stage (OR 2.31 and 3.22), and Surgical Complexity Score (SCS) (OR 1.16 and 1.23) were the clinical factors that were most substantially associated to the incidence of overall and severe complications, respectively. The resulting nomograms demonstrated great internal discrimination, good consistency, and stable calibration, with C-index of 0.74 and 0.78 for overall and severe complications prediction, respectively. A satisfactory external discrimination was also indicated by the extrapolation cohort, with the C-index for predicting overall and severe complications being 0.92 and 0.91, respectively.

**Conclusions:**

The risk of considerable postoperative morbidity exists after cytoreductive surgery for AEOC. These two nomograms with good discrimination and calibration might be useful to guide clinical decision-making and help doctors assess the probability of postoperative complications for AEOC patients.

## Introduction

Treatment for patients with advanced epithelial ovarian cancer (AEOC) includes aggressive cytoreductive surgery followed by platinum-based chemotherapy (1). As we all know, complete resection leaving no residual macroscopic tumor is a significant prognostic factor in AEOC surgery, which could also greatly increase perioperative morbidity (2). However, not all patients are able to withstand the perioperative complications following maximal cytoreductive surgery, particularly those with a poor physical status, comorbidities, or a high tumor burden (3, 4). Some researchers have already drawn attention to the contradictions between patients’ physical tolerance and tumor resectability, whatever the timing of the procedure, primary or interval debulking surgery. Furthermore, earlier research found that advanced age, advanced International Federation of Gynecology and Obstetrics (FIGO) stage, and the number of cytoreductive procedures were all independently related with an increased risk of morbidity and mortality (5–9). It is well recognized that postoperative complications can lengthen hospital stays and delay the administration of adjuvant chemotherapy, as well as negatively affect the quality of life and potentially reduce overall survival of AEOC patients (10). A recent meta-analysis showed that neoadjuvant chemotherapy (NACT), compared to primary debulking surgery (PDS), was associated with less morbidity and no difference in overall or progression free survival in advanced ovarian cancer (11). Therefore, identifying which patient subgroups might benefit from alternative treatment options such as NACT therefore requires weighing the survival benefit of maximally complete resection against its associated postoperative complications and making a preoperative prediction of the risk of postoperative morbidity.

However, there have only been a few studies that have reported predictive models for postoperative morbidity or mortality in AEOC surgery with limited evidences (12, 13). The goal of this study is to determine the influencing factors of unfavorable postoperative outcomes for AEOC patients by multicenter retrospective cohort study, and to develop and extrapolate a nomogram for predicting the risk of postoperative complications.

## Methods

### Patients

**Figure 1:**
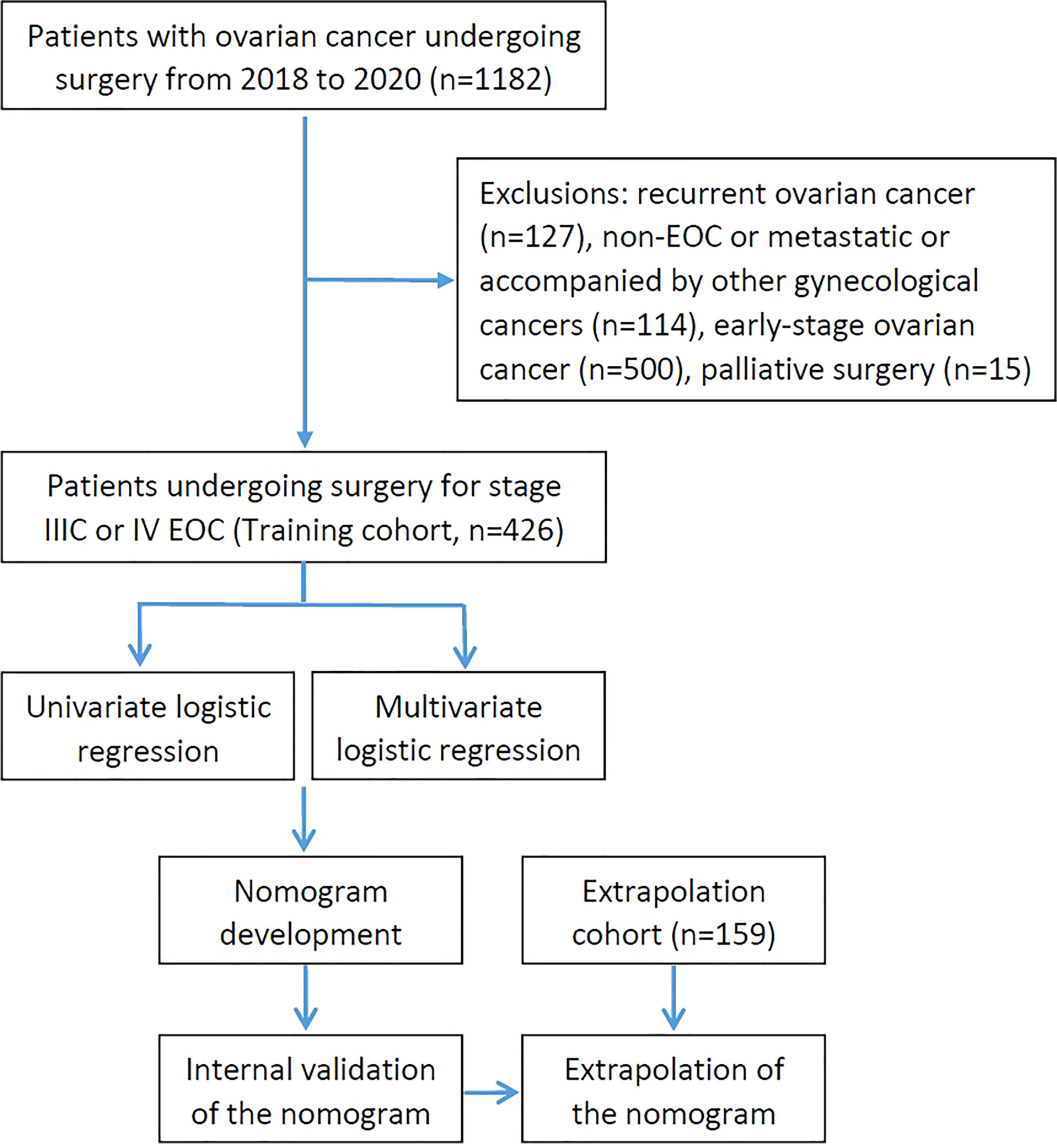
Flow chart of the overall study.

Patients who presented to West China Second University Hospital of Sichuan University with a chief complaint of a suspected ovarian mass or ovarian cancer and underwent surgery between January 1, 2018 to December 31, 2020 were retrospectively enrolled for primary screening. Patients who underwent cytoreductive surgery for stage IIIC or IV EOC (including primary peritoneal carcinoma and fallopian tube cancer) were included in this study. And patients with the following characteristics were excluded: recurrent ovarian cancer, non-EOC, early-stage ovarian cancer, palliative surgery, and accompanied by other gynecological cancers. After screening according to the inclusion and exclusion criteria, 426 patients were enrolled into the training group to establish the nomogram. Additionally, EOC patients who met the requirements from The First Affiliated Hospital of Chongqing Medical University were used for the nomogram’s extrapolation. The treatment decision-making for them was based on the Suidan criteria combined with the Fagotti criteria (14, 15). Patients with a low likelihood of a complete resection were clinically referred to NACT for subsequent interval debulking surgery (IDS), while PDS was performed on the others. The flow chart of the study was shown in **Figure 1**.

### Clinical data extraction

Clinical data were obtained from electronic medical record review and telephone follow-up. The modified frailty index (mFI) was used to assess patient’s performance status, calculated by adding one point for each of the 11 following individual variables: diabetes mellitus, functional status index of 2 or higher, chronic obstructive pulmonary disease or current pneumonia, congestive heart failure, previous myocardial infarction, previous percutaneous coronary intervention or coronary surgery or angina, hypertension requiring medication, peripheral vascular disease or resting pain, impaired sensorium, transient ischemic attack or cerebrovascular accident, and cerebrovascular disease with deficit (16). The complexity of surgical procedures was calculated postoperatively based upon the Surgical Complexity Score (SCS), according to Aletti et al (12). We reviewed the intraoperative information from electronic medical record to get the score. An ordinal scale was generated, so that patients have been stratified into 3 groups of surgical complexity: low (score ≤ 3), intermediate (score 4-7) and high (score ≥ 8). Residual disease (RD) after surgery was classified into three categories: R0 if there was no macroscopic residual disease, measurable or R1 (RD 0.1–1.0 cm), and suboptimal or R2 (RD > 1 cm) based on the largest residual tumor diameter. Besides, R0 and R1 was added together to define optimal debulking. Postoperative 30-day complications were graded using the modified Accordion classification 0-4 scale as in our previous study (17, 18), severe complications were defined as the occurrence of at least one of the following events within 30 days after surgery: unplanned readmission or reoperation, septic shock, renal failure, major cardiac events, thoracentesis, high-risk pulmonary thromboembolism, respiratory failure, or severe pneumonia. And the 30-day complications, 90-day mortality and the reasons why patients were unable to undergo adjuvant chemotherapy after surgery were asked by telephone follow-up. Continuous variables were roughly categorized by median for surgical outcomes.

### Extrapolation and statistical analyses

The normality test of the measurement data was conducted by using the Kolmogorov-Smirnov and the Shapiro-Wilks test. Continuous variables were described in terms of mean (SD) or median (quartile), and categorical variables were presented as numbers and proportions. Comparisons between groups were evaluated using Chi-squared test or Fisher exact test for categorical variables. Univariate logistic regressions analysis were performed to evaluate the association between predicting variables and 30-day postoperative severe complications and overall complications. Variables with P ≤ 0.10 were incorporated into multivariate analyses to find out the independent predicting variables. Estimates were given as odds ratios (ORs) and 95% confidence intervals (CIs). A nomogram was developed based on the most valuable predictive factors, and validation was performed using the bootstrapping correction technique. In order to prevent overestimation and to achieve a better model performance, variables with P ≤ 0.10 were entered into our prognostic model (19). A nomogram’s discrimination is measured *via* a C-index. With a binary outcome, the C-index is identical to the area under the curve (AUC) for a receiver operating characteristic (ROC) curve. The Hosmer-Lemeshow (H-L) goodness of fit test and calibration curves were used to show predicted nomogram probabilities compared to the actual probability across the range of model predictions. All analyses were conducted using the SPSS version 22.0 for Windows (SPSS Inc., Chicago, IL, USA) and R version 4.1.3 with the rms packages (https://www.r-project.org/). Statistical tests were 2-sided, with data followed by *P* < 0.05 considered statistically significant.

## Results

A total of 1182 consecutive patients with ovarian cancer undergoing surgery were recruited for primary screening from January 2018 to December 2020. As shown in **Figure 1**, 426 patients were evaluated after 756 patients were excluded in the training cohort, besides, 159 patients were enrolled in the extrapolation cohort. All IDS patients had NACT for 3 to 6 cycles without having the hyperthermic intraperitoneal chemotherapy (HIPEC) procedure. Baseline and operative characteristics of patients in the training cohort are summarized in **Table 1**. Overall, all of the patients had between 0 and 4 comorbidities, and they were divided into groups based on their mFI scores. In particular, 30 patients (7.04%) had diabetes mellitus, 78 patients (18.31%) had hypertension requiring medication, 71 patients (16.67%) had peripheral vascular disease or resting pain, 40 patients (9.39%) suffered from chronic obstructive pulmonary disease and others had previous percutaneous coronary intervention or coronary surgery or angina, cerebrovascular accident or functional status index ≥ 2. In addition, patients who received IDS (57.98%) were slightly more than PDS (42.02%). Briefly, most patients (66.67%) in this study underwent intermediate complexity surgery, and less than 20% of patients underwent either low or high complexity surgery. Specifically, patients who underwent oophorectomy with or without hysterectomy were included. 409 patients (96.01%), 308 patients (72.30%) and 271 patients (63.62%) had omentectomy, pelvic lymphadenectomy and paraaortic lymphadenectomy, respectively. 6 patients (1.41%) and 7 patients (1.64%) underwent liver and diaphragm surgery, respectively. Small bowel resection was performed in 8 patients (1.88%), whereas large bowel resection with T-T anastomosis was necessary in 55 patients (12.91%). Furthermore, 70 patients (16.43%) and 6 patients (1.41%) had colon/rectosigmoid resection and spleen surgery, respectively. Additionally, most patients (76.06%) achieved optimal debulking, whereas less than one-fourth of patients underwent suboptimal resection.

**Table 1:** Patients characteristics in training cohort.

	Total, N=426 (%)
**Age (years), mean (SD)**	54.87 (10.03)
**BMI (kg/m^2^), mean (SD)**	22.42 (2.90)
**Preoperative ASA score**	
** 2**	333 (78.17)
** 3-4**	93 (21.83)
**mFI**	
** 0-1**	358 (84.04)
** 2-4**	68 (15.96)
**Preoperative albumin (g/dL)**	
** <3.5**	14 (3.29)
** ≥3.5**	412 (96.71)
**Preoperative CA125 (U/ml), median (quartile)**	187.40 (48.53, 639.15)
**Volume of ascites (ml)**	
** ≤500**	328 (77.00)
** 500-2000**	45 (10.56)
** ≥2000**	53 (12.44)
**FIGO stage**	
** IIIC**	379 (88.97)
** IV**	47 (11.03)
**Histology**	
** Serous**	384 (90.14)
** Non-Serous**	42 (9.86)
**Surgery timing**	
** PDS**	179 (42.02)
** IDS**	247 (57.98)
**SCS**	
** low**	85 (19.95)
** intermediate**	284 (66.67)
** high**	57 (13.38)
**RD**	
** R0**	204 (47.89)
** optimal (≤1 cm)**	120 (28.17)
** suboptimal (>1cm)**	102 (23.94)
**Operative time (minutes), median (quartile)**	302.50 (235.00, 385.00)
**Intraoperative blood loss (ml), median (quartile)**	525.00 (300.00, 1000.00)
**Postoperative hospital stay(days), median (quartile)**	9 (7, 13)
**Postoperative complications**	
** mild**	53 (12.44)
** moderate**	44 (10.33)
** severe**	26 (6.10)
**90-Day mortality**	6 (1.41)
**Adjuvant chemotherapy**	
** Yes, started within 42 days**	367 (86.15)
** No, or started after 42 days**	51 (11.97)
** Unknown**	8 (1.88)

BMI, body mass index; ASA, American Society of Anesthesiologists; mFI, modified frailty index; FIGO, International Federation of Gynecology and Obstetrics; PDS, primary debulkingsurgery; IDS, interval debulking surgery; SCS, Surgical Complexity Score; RD, residual disease.

As the most vital evaluation parameters of surgical outcome in this study, we found that 123 patients (28.87%) suffered from at least one 30-day postoperative overall complications, and among them, 26 patients (6.10%) had severe complications based on the modified Accordion classification, which including 7 cases of severe pneumonia, 3 cases of thoracentesis, 9 cases of high-risk pulmonary thromboembolism, 1 case of septic shock, 1 case of respiratory failure, 3 cases of unplanned readmission and 2 cases of reoperation. The rest 97 cases of mild and moderate complications were composed of common pneumonia, wound/stoma/peritoneal cavity infections, deep vein thrombosis, moderate-risk pulmonary thromboembolism, hypohepatia, incomplete bowel obstruction and total parenteral nutrition. Besides, 90-day postoperative mortality occurred in 1.41% (6/426), which including 2 cases of bowel obstruction, 2 cases of high-risk pulmonary thromboembolism, 1 case of respiratory failure and 1 case of septic shock. Additionally, the majority of patients (367/426, 86.15%) started and finished platinum-based standard chemotherapy within 42 days of surgery, however 51 patients (11.97%) were unable to do so because of their poor physical condition. Besides, 8 patients were lost to follow-up.

**Figure 2:**
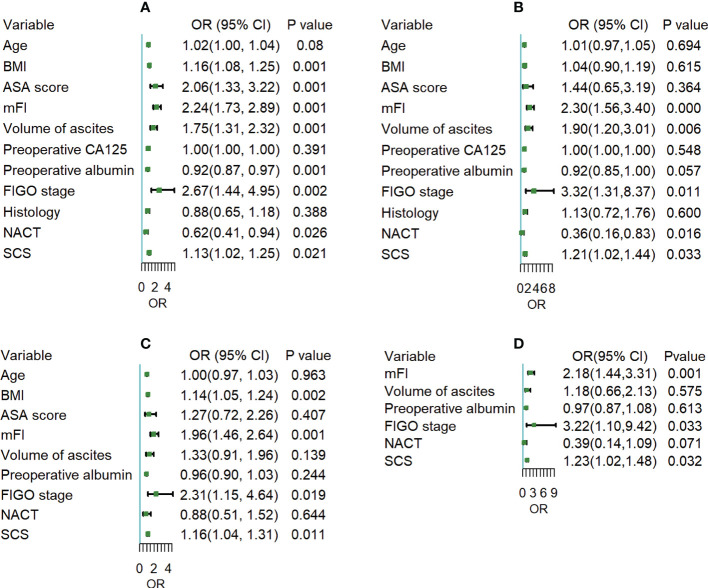
**(A, C)** Univariate and multivariate logistic regression analysis for the odds of 30-day postoperative overall complications; **(B, D)** Univariate and multivariate logistic regression analysis for the odds of 30-day postoperative severe complications.

We first examined the factors underlying 30-day postoperative overall complications. BMI, ASA, mFI, volume of ascites, preoperative albumin, FIGO stage, NACT and SCS were all correlated with overall complications in univariate analysis. In multivariate analysis, BMI (odds ratio [OR] 1.14; 95% confidence interval [CI] 1.05 to 1.24), mFI (OR 1.96; 95% CI 1.46 to 2.64), FIGO stage (OR 2.31; 95% CI 1.15 to 4.64), and SCS (OR 1.16; 95% CI 1.04 to 1.31) were independent predictors of overall complications. Then, for 30-day postoperative severe complications, univariate analysis showed a significant association between mFI, volume of ascites, preoperative albumin, FIGO stage, NACT, SCS and occurrence of severe complications. At multivariate analysis, mFI (OR 2.18; 95% CI 1.44 to 3.31), FIGO stage (OR 3.22; 95% CI 1.10 to 9.42), and SCS (OR 1.23; 95% CI 1.02 to 1.48) were independent predictors of severe complications. In summarizing, whatever postoperative overall or severe complications, the most significant contributors were FIGO stage and mFI, followed by SCS, as shown in **Figure 2**.

Additionally, we grouped the patients in the training cohort by overall and severe complications according to the influencing variables and surgical outcomes, as shown in **Table 2**. Overall complications were more likely to occur in patients with higher BMI, mFI, FIGO stage, SCS, and who had not received NACT (*P* < 0.05). For severe complications, higher mFI, FIGO stage, SCS, and who had not received NACT all increased the risks of severe complications (*P* < 0.05). There were significantly higher percentages of patients with operative time not less than 300 minutes, intraoperative blood loss not less than 500 ml, and postoperative hospital stays not less than 9 days in the overall and severe complications populations (*P* < 0.05).

**Table 2:** Comparisons of the overall and severe complications between the groups.

		Overall complications, N=123 (%)	*P* value	Severe complications, N=26 (%)	*P* value
**BMI (kg/m^2^)**			<0.001		0.927
** <24**	308	72 (23.38)		19 (6.17)	
** ≥24**	118	51 (43.22)		7 (5.93)	
**mFI**			<0.001		<0.001^*^
** 0-1**	358	84 (23.46)		14 (3.91)	
** 2-4**	68	39 (57.35)		12 (17.65)	
**FIGO stage**			0.001		0.016^*^
** IIIC**	379	100 (26.39)		19 (5.01)	
** IV**	47	23 (48.94)		7 (14.89)	
**Surgery timing**			0.025		0.013
** PDS**	179	62 (34.64)		17 (9.50)	
** IDS**	247	61 (24.70)		9 (3.64)	
**SCS**			0.009		0.027
** low**	85	25 (29.41)		4 (4.71)	
** intermediate**	284	72 (25.35)		14 (4.93)	
** High**	57	26 (45.61)		8 (14.04)	
**Operative time (minutes)**			<0.001		0.027
** <300**	204	43 (21.08)		7 (3.43)	
** ≥300**	222	80 (36.04)		19 (8.56)	
**Intraoperative blood loss (ml)**			<0.001		0.002
** <500**	171	33 (19.30)		3 (1.75)	
** ≥500**	255	90 (35.29)		23 (9.02)	
**Postoperative hospital stay(days)**			<0.001		<0.001
** <9**	186	9 (4.84)		2 (1.08)	
** ≥9**	240	114 (47.50)		24 (10.00)	
**Adjuvant chemotherapy**			0.267		0.258
** Yes, started within 42 days**	367	102 (27.79)		21 (5.72)	
** No, or started after 42 days**	51	18 (35.29)		5 (9.80)	

*fisher test.

**Figure 3:**
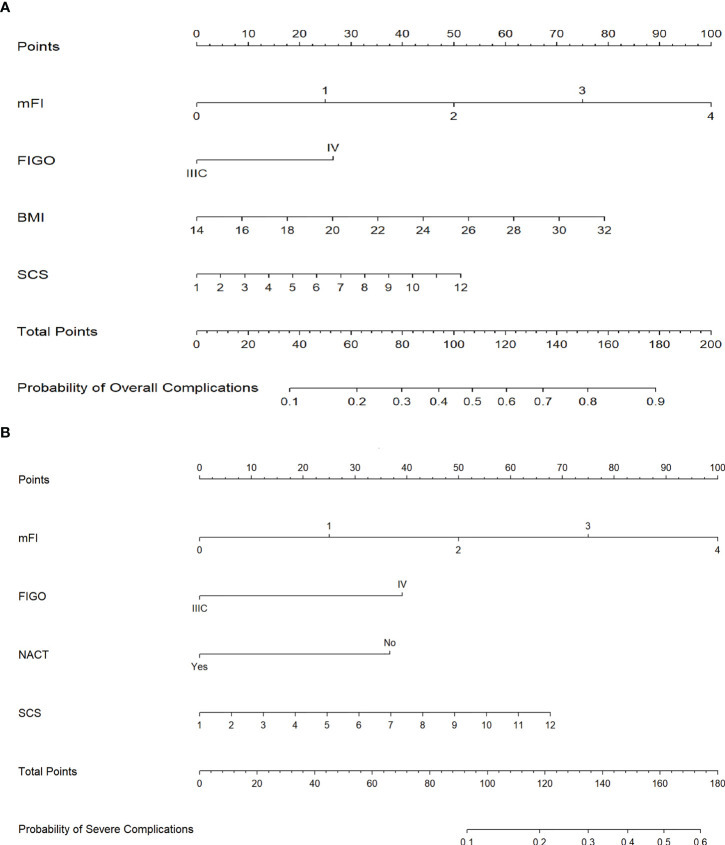
**(A)** Nomogram for prediction of 30-day postoperative overall complications. **(B)** Nomogram for prediction of 30-day postoperative severe complications. (Annotation: Points are assigned for each of the patient characteristics by drawing a line up from the scale for each predictor to the points bar at the top of the figure. The points for all predictors are then added to determine the total points. A patient’s predicted probability of 30-day postoperative overall or severe complications is determined by drawing a line from the total points bar to the predicted probability bar.).

We used the best influencing variables from the multivariate analysis to construct nomograms for predicting postoperative overall and severe complications. Using the nomograms, points are added accordingly to each weighted preoperative variable and the point total corresponds to a predicted probability of overall complications (**Figure 3A**) or severe complications (**Figure 3B**).

**Figure 4:**
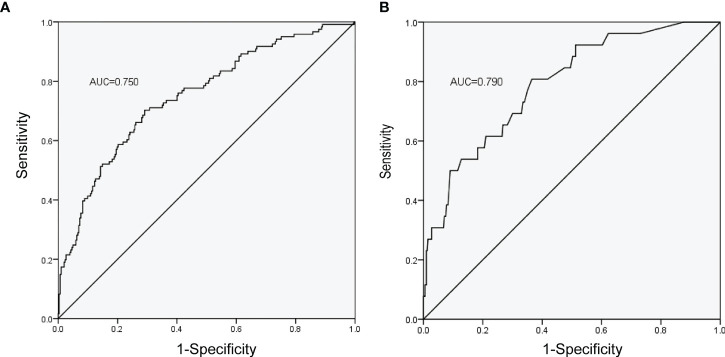
ROC curves of the nomograms with the training cohort. **(A)** Nomogram for prediction of overall complications. **(B)** Nomogram for prediction of severe complications.

**Figure 5:**
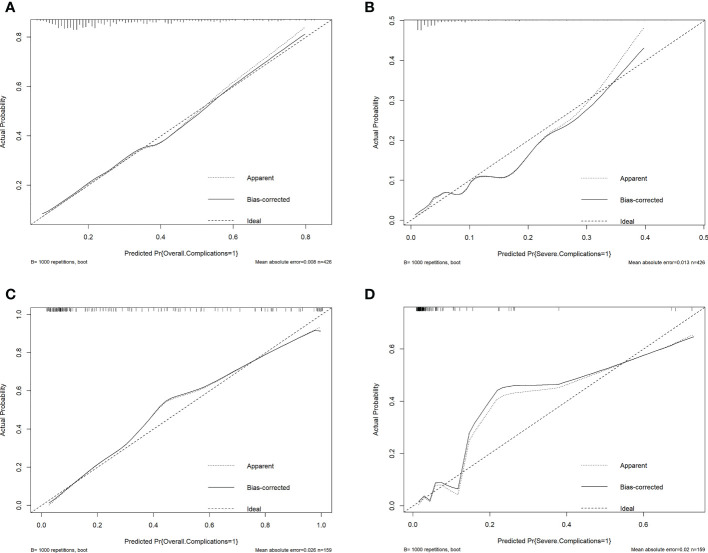
Validation plots for evaluating nomogram calibration (1). Internal validation in the training cohort. **(A)**, Nomogram for prediction of overall complications. **(B)**, Nomogram for prediction of severe complications. (2) Extrapolation in the extrapolation cohort. **(C)**, Nomogram for prediction of overall complications. **(D)**, Nomogram for prediction of severe complications.

The ROC curves of the nomograms with internal validation were shown in (**Figures 4A, B**). The models had good distinction as demonstrated by the AUC values of 0.75 (95% CI 0.70-0.80, *P* < 0.001) and 0.79 (95% CI 0.70-0.88, *P* < 0.001) for predicting overall and severe complications, respectively. Meanwhile, the bootstrap-corrected C-index were 0.74 (95% CI 0.68-0.79) and 0.78 (95% CI 0.69-0.87) for each model, indicating good consistency between the prediction and actual observation. Internal validation of the nomograms showed that the calibration plots are close to the 45° line, which indicates good calibration (**Figures 5A, B**).

Lastly, we made an extrapolation of the aforementioned nomograms using the medical records of 159 AEOC patients from The First Affiliated Hospital of Chongqing Medical University. The calibration plots of the nomograms with extrapolation were shown in (**Figures 5C, D**). The C-index were 0.92 (95% CI 0.87-0.96) and 0.91 (95% CI 0.80-1.00) for predicting overall and severe complications, respectively, indicating that the models had good external discrimination. Additionally, extrapolation of the nomograms showed that the calibration plots are near the 45° line, which suggests acceptable calibration.

## Discussion

Overall, this multicenter retrospective cohort study develops the model to predict the complications after cytoreductive surgery (including PDS and IDS) in AEOC and extrapolates it. Firstly, we found that BMI, mFI, FIGO stage, and SCS were independent predictors of overall complications, and for severe complications, the independent predictors were mFI, FIGO stage, NACT, and SCS. The most significant contributors for both overall complications and severe complications, in particular, were FIGO stage and mFI, which were then followed by SCS, BMI or NACT. Secondly, inter-group comparisons revealed that patients with higher mFI, FIGO stage, SCS, and upfront PDS had a higher probability of having overall and severe complications. Moreover, patients may be more likely to suffer subsequent negative surgical outcomes in the groups with overall and severe complications. Thirdly, we built two nomograms for the 30-day postoperative overall complications and severe complications, respectively. Strong internal calibration was also indicated by the C-index of 0.74 (AUC 0.75) and 0.78 (AUC 0.79), respectively, for each model. These values showed good distinction and consistency between the prediction and actual observation, as well as good calibration as shown in the calibration plots. Fourthly, extrapolation was performed well in the extrapolation cohort of patients undergoing cytoreduction. All of the results we summarized above robustly confirmed that higher mFI, FIGO stage, SCS, and upfront PDS were significantly positively correlated with overall and severe complications. The nomograms created by these influencing factors might be effective in predicting overall and severe complications for AEOC patients, and also assisting surgeons in making the most appropriate clinical decisions.

Over 70% of ovarian cancer patients are diagnosed at an advanced stage. 11.03% of patients were diagnosed with FIGO IV stage in our study. Advanced stage means a higher tumor load and, consequently, a more complex surgical procedure, increasing the risk of postoperative complications. According to our findings, the training cohort suffered an incidence of postoperative overall and severe complications of 28.87% and 6.10%, respectively. Kumar et al. have reported that FIGO stage and surgical complexity were positively associated with postoperative severe complications following PDS in advanced ovarian cancer (10). Our findings were consistent with the recent study. We observed that compared to the IIIC stage, AEOC patients with the IV stage have about 2-fold and 3-fold risks of overall complications and severe complications, respectively. Accumulating studies have shown that R0 is the ultimate goal of cytoreduction surgery in AEOC. We also noted that AEOC itself is a severe condition with a high morbidity and mortality rate in the perioperative period. Therefore, the risk of adverse postoperative outcomes associated with maximal cytoreduction surgery should be weighed against its survival benefit (3, 17). Here, our present study had 80.05% of patients undergoing intermediate or high complexity surgery, and 76.06% of patients achieving optimal debulking. And we found that, as an independent predictor, SCS was positively correlated with the occurrence of postoperative overall (OR 1.16) complications and severe (OR 1.23) complications. The timing of surgery for AEOC patients is a longstanding controversy within the gynecologic oncology academic community (20, 21). Some gynecologic oncologists hold that upfront complete cytoreduction should remain the standard of care for advanced ovarian cancer. However, the others insisted that for non-operable patients or those with a low likelihood of achieving complete cytoreduction, NACT is preferred, as it is associated with lower postoperative morbidity and mortality (22). It is critical and challenging to screen out fit patients to receive suitable treatment. Our study showed that patients who received NACT followed by IDS (57.98%) were slightly more than PDS (42.02%). In addition, inter-group analysis showed that patients who had not received NACT were more likely to suffer overall and severe complications. In other words, PDS was negatively correlated with postoperative complications. This is consistent with recent publications (11, 22). Based on the Canadian Study of Health and Aging Frailty Index, the mFI has been already validated that is useful in the preoperative risk assessment and prediction of outcomes in patients undergoing gynecological surgery (16). Our results suggest that mFI is an independent predictor of both overall (OR 1.96) and severe (OR 2.18) complications (23). Besides, we further observed that BMI ≥ 24 could independently predict overall complications. Understandably, the mild complications in this study were mostly made up of wound infection and deep vein thrombosis, which can be mainly attributed to being overweight. Additionally, we also found that patients with overall or severe complications were more likely to suffer other adverse surgical outcomes, including more intraoperative blood loss, being unable to undergo planned adjuvant chemotherapy, longer operative time, and postoperative hospital stays.

At present, although there are a few models predicting the postoperative complications of ovarian cancer, there are also some limitations. Kumar et al (10) have demonstrated that age, albumin < 3.5 g/dL, surgical complexity, stage, ASA, and BMI influence morbidity and mortality after debulking surgery. But only PDS patients were included in the study, so it lacks NACT as a factor for preoperative analysis. Cham et al (13) have created a nomogram for patients who are being considered for primary debulking or neoadjuvant chemotherapy, with an internal discrimination C-index of 0.71. However, a flaw that cannot be ignored was that the researchers were unable to distinguish whether a research subject underwent primary or interval cytoreduction and the stage at the time of diagnosis. In addition, other models have used single institutional data to determine factors related to postoperative morbidity and mortality and without the establishment of a nomogram (12, 24, 25). We next used the best influencing variables from the multivariate analysis to construct nomograms for predicting postoperative overall and severe complications in this study. Our data suggested that characteristics of the disease (FIGO stage) and physical fitness and health status (mFI) were the strongest influencing factors for both overall complications and severe complications, followed by SCS, BMI or NACT. Moreover, the internal validation showed a good distinction with the C-index of 0.74 and 0.78, respectively, as well as good consistency and calibration shown in the results. In addition, the extrapolation was independently validated with a C-index comparable to the training cohort, which indicates good external discrimination. In consideration of the preoperative timeliness, we chose to focus on preoperative factors for the nomograms, to maximize clinical utility for both gynecologic oncology surgeons and patients. According to a prior study, a preoperative CT scan can be used to predict surgical complexity (10). In light of this study, when using the nomogram in clinical practice, we can first determine the approximate SCS score by evaluating planned operations through CT scan, MRI imaging, or PET-CT. Laparoscopy or a small incision to allow exploration could also be used to assess the extent and complexity of the procedure prior to cytoreductive surgery. For this select group of patients, preventive actions should be taken, or NACT could be performed rationally if broader disease was found and increased the overall score. The score needs to be validated using preoperative imaging findings to predict the surgical complexity for utilization in clinical practice. When the nomogram predicts a high risk of overall complications, such as wound infection and deep vein thrombosis, clinicians should pay attention to prevent adverse outcomes after surgery, accordingly, standardized postoperative care should include postoperative changing dressings in time, duration of antibiotics use and mechanical assisted compression therapy. Similar to this, the nomogram can benefit in the screening out of candidates for surgery who are not suitable, as well as helping clinicians and patients in choosing an appropriate treatment, when it predicts a high risk of severe complications, particularly those complications with a high fatality rate.

This study has the advantages of being multicenter and only including FIGO IIIC and IV ovarian cancer, which makes it pertinent for patients who are most at risk for surgical morbidity and mortality. Decision-making regarding their treatment is more challenging and contentious in the clinical setting because they are AEOC. The majority of research utilized a single index to predict ovarian cancer postoperative complications, however this study employed several preoperative parameters to build nomograms. Each parameter was weighted, indicating the significance of each risk factor. The total score was calculated to determine whether a patient was a candidate for upfront surgery or NACT-IDS. NACT can reduce tumor burden in patients with high tumor burdens who needed high complexity surgery, which lowers the risk of severe postoperative complications while achieving complete resection. Besides, there is internal validation and extrapolation for the nomograms, which is uncommon in other studies. According to certain researchers, selective patients identified for IDS should be provided HIPEC due to its advantages in improving patients’ survival and lack of increased risk of side effects (26, 27). Because all participants did not undergo HIPEC, there was no correlation analysis done between HIPEC and the postoperative complications. This is a limitation of our study. Another limitation of our study is the nature of its retrospective design, which is vulnerable to selection bias. Moreover, most patients underwent intermediate complexity surgery, and less than 14% of patients underwent high complexity surgery, which results in relatively low surgical risk, and this is similar to the previous study (13). Additionally, 90-day postoperative mortality in our study occurred at merely 1.41%, which is too low to do statistical analysis. Extensive external validation of these two nomograms in other settings is necessary.

## Conclusion

Cytoreductive surgery for AEOC is at significant increased risk for the occurrence of substantial postoperative morbidity. This study suggests several influencing factors for postoperative complications for AEOC patients *via* a multicenter retrospective cohort study, as well as builds and extrapolates the nomograms for predicting the risk of postoperative complications. Additionally, the SCS score needs to be validated using preoperative imaging findings to predict the surgical complexity for utilization in clinical practice. These two models play a significant role in risk stratification and clinical decision-making for AEOC patients. Broader validation of the model in various surgical settings is necessary.

## Data availability statement

The original contributions presented in the study are included in the article/supplementary material. Further inquiries can be directed to the corresponding authors.

## Author contributions

The work reported in the paper has been performed by the authors, unless clearly specified in the text. CJ collected and statistically analyzed the medical records, as well as wrote the manuscript. YL and JT collected the medical records. ZL and WM provided practical suggestions and critically revised the manuscript. All authors contributed to the article and approved the submitted version.

## Funding

The Department of Science and Technology of Sichuan Province (grant number 21PJ050).

## Acknowledgments

We sincerely thank for the support by the Sichuan Province Science and Technology Support Program (grant number 21PJ050).

## Conflict of interest

The authors declare that the research was conducted in the absence of any commercial or financial relationships that could be construed as a potential conflict of interest.

## Publisher’s note

All claims expressed in this article are solely those of the authors and do not necessarily represent those of their affiliated organizations, or those of the publisher, the editors and the reviewers. Any product that may be evaluated in this article, or claim that may be made by its manufacturer, is not guaranteed or endorsed by the publisher.
